# The antiprotozoal drug pentamidine ameliorates experimentally induced acute colitis in mice

**DOI:** 10.1186/1742-2094-9-277

**Published:** 2012-12-23

**Authors:** Giuseppe Esposito, Elena Capoccia, Giovanni Sarnelli, Caterina Scuderi, Carla Cirillo, Rosario Cuomo, Luca Steardo

**Affiliations:** 1 Department of Physiology and Pharmacology ‘Vittorio Erspamer’, University SAPIENZA of Rome, P. le Aldo Moro 5, 00185, Rome, Italy; 2 Department of Clinical and Experimental Medicine, University of Naples FEDERICO II, Via S. Pansini 5, 80131, Naples, Italy; 3 Current address: Laboratory for Enteric NeuroScience (LENS), TARGID, KU Leuven, Herestraat 49, 3000, Leuven, Belgium

**Keywords:** Pentamidine, Acute colitis, S100B protein, Enteric glia

## Abstract

**Background:**

Intestinal inflammation is partly driven by enteroglial-derived S100B protein. The antiprotozoal drug pentamidine directly blocks S100B activity. We aimed to investigate the effect of pentamidine on intestinal inflammation using an animal model of dextran sodium sulphate (DSS)-induced acute colitis.

**Methods:**

Mice were divided into: control group, colitis group (4% DSS for four days) and two pentamidine-treated colitis groups (0.8 mg/kg and 4 mg/kg). Anti-inflammatory effect of pentamidine was assessed in colonic tissue by evaluating the disease activity index and the severity of histological changes. Colonic tissue were also used to evaluate cyclooxigenase-2, inducible nitric oxide synthase, S100B, glial fibrillary acidic protein, phosphorylated-p38 MAPkinase, p50, p65 protein expression, malondyaldheyde production, mieloperoxidase activity, and macrophage infiltration. Nitric oxide, prostaglandin E_2_, interleukin-1 beta, tumor necrosis factor alpha, and S100B levels were detected in plasma samples. Parallel measurements were performed *in vitro* on dissected mucosa and longitudinal muscle myenteric plexus (LMMP) preparations after challenge with LPS + DSS or exogenous S100B protein in the presence or absence of pentamidine.

**Results:**

Pentamidine treatment significantly ameliorated the severity of acute colitis in mice, as showed by macroscopic evaluation and histological/biochemical assays in colonic tissues and in plasma. Pentamidine effect on inflammatory mediators was almost completely abrogated in dissected mucosa but not in LMMP.

**Conclusions:**

Pentamidine exerts a marked anti-inflammatory effect in a mice model of acute colitis, likely targeting S100B activity. Pentamidine might be an innovative molecule to broaden pharmacological tools against colitis.

## Background

Although the etiology of ulcerative colitis (UC) remains incompletely understood, severe and persistent mucosal infiltration of macrophages and neutrophils in the large intestine represents a prominent feature [1]. Immune cells release cytokines, interleukins and proinflammatory signaling molecules [2-4]. In addition to the well-known involvement of macrophages and neutrophils, other cell types have been recently reported to substantially contribute to the onset and progression of the disease. Enteric glial cells (EGC) play a fundamental role in the maintenance of gut homeostasis since they have trophic and protective functions toward enteric neurons and are fully implicated in the modulation of neuronal activities [5]. Moreover, EGC have been repeatedly reported to trigger and support intestinal inflammation [6] and to function as a first line of defense against pathogens [7]. EGC may proliferate and be activated in response to injury and inflammation undergoing reactive gliosis (enterogliosis), a condition in which they release neurotrophins, growth factors and proinflammatory cytokines cross-talking with other infiltrating immune cells [8]. Alterations in the homeostasis of the enteric nervous system are induced by reactive enterogliosis and are characterized by the massive overexpression and secretion of specific astroglial-derived signaling molecules such as S100B protein [9,10].

S100B is a diffusible, Ca^++^/Zn^++^-p53 binding protein playing a pivotal role during intestinal inflammation, since it orchestrates proinflammatory signals [11,12]. Aberrant expression and release of S100B correlate with the inflammatory status of the gut. S100B accumulates at the RAGE (receptor for advanced glycation end products) site only in micromolar concentrations [11,13-15] and such interaction leads to mitogen-activated protein kinase (MAPK) phosphorylation and consequent nuclear factor-kappaB (NF-κB) activation, which, in turn, promote the transcription of different cytokines and inducible nitric oxide synthase (iNOS) protein [14]. Molecular targeting of S100B protein during intestinal inflammation might therefore represent an innovative approach to treat UC.

Pentamidine isethionate, discovered to have antiprotozoal activity in 1938, and approved in the United States for the treatment of Pneumocystis carinii pneumonia and other protozoal diseases [16], appears to be an intriguing candidate. In addition to its antiprotozoal activity, pentamidine has been reported to inhibit S100B activity because of its ability to block the interaction at the Ca^++^/p53 site of the protein [17].

Based on this background, the present study was aimed at evaluating the beneficial effect of a daily administration of pentamidine in an acute model of UC induced by dextran sulphate sodium (DSS) administration in drinking water in CD-1 mice. DSS-induced colitis is highly reproducible and is a well-known *in vivo* model of experimental colitis in rodents that reproduces many features of UC [18]. We tested the effect of pentamidine on (i) intensity of the symptoms (diarrhea, blood in the feces, animal weight loss) through a disease activity index (DAI) scale [19]; (ii) release of cytokines and proinflammatory signaling molecules present in mice plasma; (iii) postmortem evaluation of macroscopic shortening of large intestine and spleen weight; (iv) global colonic inflammation by the evaluation of biochemical and histological changes of the tissue.

## Methods

### Animals and experimental design

**Figure 1:**
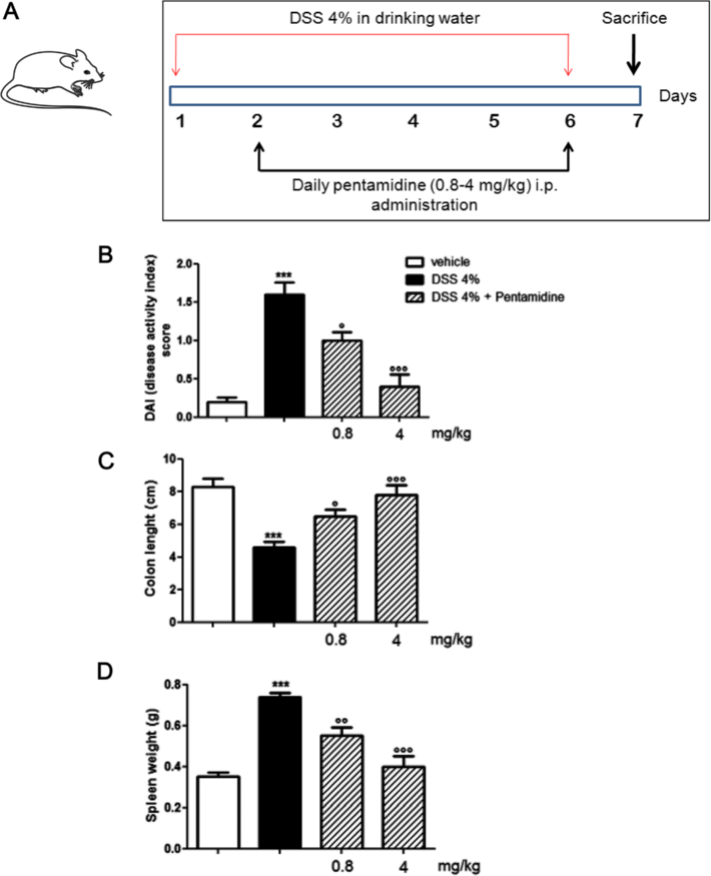
**(A) Dextran sulphate sodium (DSS)-exposed (4%) mice were treated daily with 0.8 mg/kg or 4 mg/kg pentamidine given intraperitoneally. **Effect of pentamidine on **(B) **DAI score, **(C) **colonic length and **(D) **spleen weight in DSS-treated mice. Results are expressed as mean ± SEM of *n* = 5 experiments. ***P <0.001 vs. vehicle (saline); °*P *<0.05; °°*P *<0.01 and °°°*P *<0.001 vs. DSS. DAI, disease activity index.

Six-weeks-old male CD-1 mice (25 to 35 g; Harlan Laboratories, Udine, Italy) were used for the experiments. Animals were randomly divided into five groups (n = 10 each): noncolitic control group; colitic group; colitic group receiving daily pentamidine 0.8 mg/kg; colitic group receiving daily pentamidine 4 mg/kg; noncolitic group receiving daily pentamidine 4 mg/kg (as drug internal control). Colitis was induced by administrating DSS (4% w/v, MW 36,000 to 50,000) in drinking water for six consecutive days (starting from day 1), as described in Figure 1A. Pentamidine was given intraperitoneally starting at day 2 through day 6. At day 7, animals were sacrificed and tissues were removed to perform macroscopic, histochemical and biochemical analyses as described below.

### Disease activity index (DAI)

The DAI scale is based on the evaluation of different parameters characterizing experimental colitis induction and progression. Body weight, presence of gross blood in the feces and stool consistency were recorded daily (from day 0 to 7) by an observer blinded to the treatment. According to the criteria proposed by Cooper *et al*. [19], the DAI was determined by scoring changes in: weight loss (0 = none; 1 = 1 to 5%; 2 = 5 to 10%; 3 = 10 to 20%; 4 = >20%); stool consistency (0 = normal; 2 = loose; 4 = diarrhea) and rectal bleeding (0 = normal; 2 = occult bleeding; 4 = gross bleeding). At the end of the experiment, mice were sacrificed and colons and spleens were isolated to measure the length and weight of colon and spleen, respectively.

### Preparation of cytosolic extracts and western blot analysis

Removed colonic tissues were processed for western blot analysis. Briefly, after homogenization in ice-cold hypotonic lysis buffer, protein concentration was determined using Bio-Rad protein assay kit (Bio-Rad, Milan, Italy). Analysis of cyclooxigenase (COX)-2, iNOS, TNF-α, S100B, glial fibrillary acidic protein (GFAP), phosphorylated-p38 (p-p38) MAPK, p50, p65 and β-actin protein expression was performed on total protein fractions of homogenates. Equivalent amounts (50 μg) of each homogenate underwent electrophoresis through a polyacrilamide minigel. Proteins were then transferred onto nitrocellulose membrane that were saturated by incubation with 10% nonfat dry milk in 1× PBS overnight at 4°C and then incubated with either mouse anti-S100B (1:200 v/v, Neo-Marker, Milan, Italy), mouse anti-iNOS (1:2000 v/v, BD Biosciences, Milan, Italy), rabbit anti-COX-2 (1:250 v/v, BD Biosciences), GFAP (1:5000 v/v, Abcam, Cambridge, UK), mouse anti-p50, mouse anti-p65 (1:1000 v/v, Santa Cruz Biotechnology, Santa Cruz, CA, USA), or mouse anti-β-actin (1:1,000 v/v, Santa Cruz Biotechnology) for 2 h at room temperature (RT). Membranes were then incubated with anti-mouse or anti-rabbit immunoglobulins coupled to peroxidase (1:2000 v/v, Dako, Milan, Italy). Immune complexes were revealed by using enhanced chemiluminescence detection reagents (Amersham Biosciences, Milan, Italy) and exposed to Kodak X-Omat film (Eastman Kodak Co., Rochester, NY, USA OK). Protein bands were then scanned and densitometrically analyzed with a GS-700 imaging densitometer.

### Preparation of blood samples

Before being sacrificed, mice were deeply anesthetized and the blood was taken by cardiac puncture and collected in 5% EDTA vials. To determine nitric oxide (NO), prostaglandin E2 (PGE_2_), IL-1β, TNF-α, and S100B levels, plasma was isolated from the blood, immediately frozen, and stored at −80°C until the assays.

### Plasma NO quantification

NO was measured as nitrite (NO_2_^-^) accumulation in plasma. A spectrophotometer assay based on the Griess reaction was used [20]. Briefly, Griess reagent (1% sulphanilamide, 0.1% naphthylethylenediamine in H_3_PO_4_) was added to an equal volume of plasma and the absorbance was measured at 550 nm. NO_2_^-^ concentration (nM) was thus determined using a standard curve of NaNO_2_.

### Plasma PGE_2_, TNF-α, S100B, and IL-1β quantification

Quantitative determination of PGE_2_, IL-1β, TNF-α, and S100B was carried out performing enzyme linked-immunosorbent assay (ELISA) on plasma samples (PGE_2_, TNF-α, IL-1β kits were from Invitrogen, Milan, Italy; S100B kit was from BioVendor, Heidelberg, Germany) according to the manufacturer’s kit instructions.

### Macrophage infiltration in the mucosa

Samples for immunohistochemical assessment were isolated from distal colon and fixed in 4% paraformaldehyde, embedded in paraffin, sectioned in 15 μm slices and processed for immunohistochemistry. Slices were pretreated for 20 min using heat-mediated antigen retrieval with a sodium citrate buffer, incubated with MAC387 [21] (1 μg/ml, Abcam) for 15 min at RT, and detected using horseradish peroxidase (HRP)-conjugated compact polymer system. DAB was used as the chromogen. Slices were then counterstained with hematoxylin, mounted with Eukitt and analyzed with a microscope (Nikon Eclipse 80i by Nikon Instruments Europe, Amstelveen, Netherlands). Images were captured by a high-resolution digital camera (Nikon Digital Sight DS-U1).

### p53 Immunofluorescence

Colon slices derived from both control and treated mice were blocked in 10% bovine serum albumin 0.1% Triton-PBS solution for 90 min at RT and subsequently stained for 1 h at RT with anti-p53 antibody (1:1250, Santa Cruz Biotechnology) and anti-MAC387 (1:200, Abcam). Sections were then incubated for 1 h at RT in the dark with the proper secondary antibody: fluorescein isothiocyanate-conjugated anti-rabbit (1:100, Abcam) or Texas Red-conjugated anti-mouse (1:64, Abcam), respectively. Nuclei were stained with Hoechst (1:5000, Sigma-Aldrich, Milan, Italy). Slides were thus analyzed with a microscope (Nikon Eclipse 80i by Nikon Instruments Europe), and images were captured at 10X and 20X magnification by a high-resolution digital camera (Nikon Digital Sight DS-U1).

### Myeloperoxidase (MPO) assay

MPO, a marker of polymorphonuclear leukocyte accumulation and general inflammation occurring in colonic tissue, was determined as previously described [22]. After removal, colonic tissue was rinsed with cold saline, opened and deprived from the mucosa using a glass slide. The resulting layer was then homogenized in a solution containing 0.5% hexadecyltrimethylammonium bromide dissolved in 10 mM potassium phosphate buffer (pH 7.0) and centrifuged for 30 min at 20000 × g at 37°C. An aliquot of the supernatant was mixed with a solution of tetramethylbenzidine (1.6 mM) and 0.1 mM H_2_O_2_. The absorbance was then spectrophotometrically measured at 650 nm. MPO activity was determined as the amount of enzyme degrading 1 mmol of peroxide per minute at 37°C and was expressed in milliunits per 100 mg of wet tissue weight.

### Lipid peroxidation assay

Malonyl dialdehyde (MDA) was measured by the thiobarbituric acid colorimetric assay [23] in colonic tissue. Briefly, 1 mL 10% (w/v) trichloroacetic acid was added to 450 μl of tissue lysate. After centrifugation, 1.3 mL 0.5% (w/v) thiobarbituric acid was added and the mixture was heated at 80°C for 20 min. After cooling, MDA formation was recorded (absorbance 530 nm and absorbance 550 nm) in a Perkin Elmer (Waltham, MA, USA) spectrofluorimeter and the results were presented as ng MDA/mL.

### Mucosa and LMMP preparations

In order to further evaluate the putative site and the mechanism of action of pentamidine, additional experiments were set up as follows. Colonic segments (approximately 1.5 cm long) were isolated from another set of CD-1 mice not previously used for any *in vivo* treatment. Mice were euthanized by injection of pentobarbital sodium (100 mg/kg) and distal colon was removed and cut longitudinally to expose the mucosa. Under sterile conditions, the tissues were placed in Dulbecco’s modified Eagle's medium (supplemented with 5% fetal bovine serum, 2 mM glutamine, 100 U/mL penicillin, 100 μg/mL streptomycin, all from Invitrogen), pinned flat and the mucosa was carefully peeled off to obtain the longitudinal muscle-myenteric plexus (LMMP) layer [24]. Depending upon the experimental plan, both mucosa and LMMP were stimulated for 24 h with exogenous lipopolysaccharide (LPS) (10 μg/ml) + DSS (1% w/v) or exogenous S100B (5 μM, Sigma-Aldrich, Milan, Italy), with or without the addition of pentamidine (0.5 to 5 μM) administered to the tissue 10 min prior to LPS + DSS or S100B stimulus.

### Statistical analysis

Results were expressed as mean ± SEM of experiments. Statistical analysis was performed using parametric one-way analysis of variance (ANOVA) and multiple comparisons were performed by Bonferroni's post hoc test. *P* values <0.05 were considered significant.

## Results

### Pentamidine ameliorates DAI score, preserves colonic length and reduces splenomegaly induced by DSS

Starting from day 4 after DSS administration, DAI score was significantly increased in DSS groups. As expected, DSS caused a consistent increase in bloody diarrhea together with loss of body weight, as compared to control group (Figure 1B). DSS also caused a significant shortening of colon and a marked increase of spleen weight (Figure 1C and D). Pentamidine treatment inhibited the rise in DAI score in a dose-dependent way, suggesting an overall improvement of intestinal symptoms associated with colitis after DSS administration; such effect was accompanied by a reduction of bloody stools, diarrhea frequency, and a rescue of body weight (Figure 1B). Moreover, pentamidine was able to preserve colonic length and to prevent splenomegaly in DSS-treated mice (Figure 1C and D).

### Pentamidine inhibits intestinal inflammation induced by DSS

**Figure 2:**
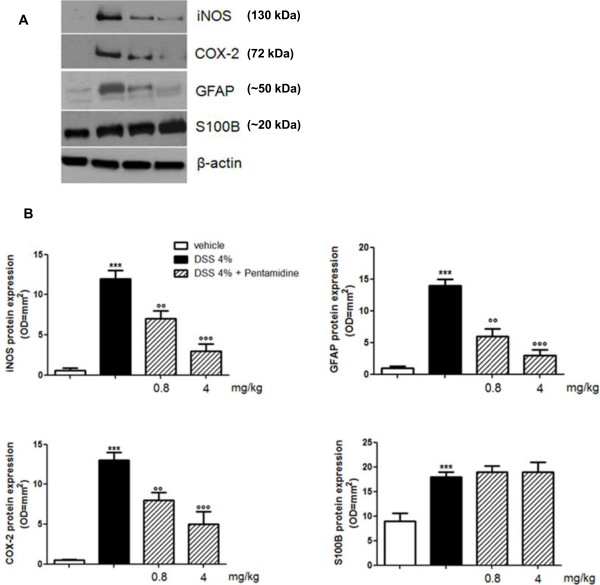
**(A) Western blot analysis showing the effect of pentamidine on iNOS, COX-2, GFAP and S100B protein expression in colonic tissue of DSS-treated mice; (B) relative quantification of immunoreactive bands (arbitrary units). **Results are expressed as mean ± SEM of *n* = 5 experiments performed in triplicate. ***P <0.001 vs. vehicle (saline); °°*P* <0.01 and °°°*P *<0.001 vs. DSS. COX2, cyclooxigenase-2; DSS, dextran sodium sulphate; GFAP, glial fibrillary acidic protein; iNOS, inducible nitric oxide synthase.

**Figure 3:**
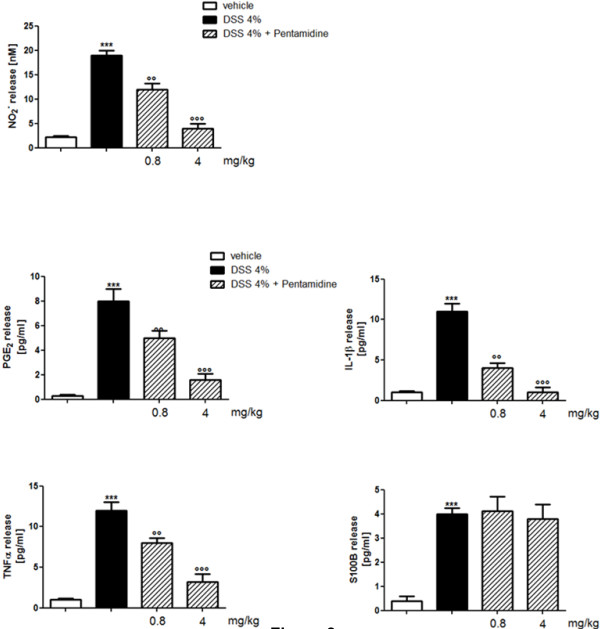
**Effect of pentamidine on the release of inflammatory mediators (NO**_**2**_^**-**^**, PGE**_**2**_**, IL-1β, TNF-α) and S100B in plasma of DSS-treated mice.** Results are expressed as mean ± SEM of *n* = 5 experiments performed in triplicate. ***P <0.001 vs. vehicle (saline); °°*P *<0.01 and °°°*P *<0.001 vs. DSS. DSS, dextran sodium sulphate; IL-1β, interleukin-1 beta; NO, nitric oxide; PGE2, prostaglandin E2; TNF-α, tumor necrosis factor alpha.

In Figure 2 it is shown that the administration of DSS caused a marked increase of iNOS, COX-2, S100B and GFAP protein expression compared to control mice. Pentamidine treatment resulted in a dose-dependent attenuation of iNOS, COX-2 and GFAP but not of S100B protein overexpression (Figure 2A and B). As expected, Griess reaction and ELISA demonstrated that the administration of DSS caused a significant increase in plasma NO_2_^-^ level, PGE_2_, IL-1β, TNF-α and S100B, compared to control mice (Figure 3). Also in this case and according to western blot results, pentamidine treatment caused a marked and dose-dependent attenuation of all the inflammatory mediators in plasma, except for S100B release that remained unaffected after pentamidine treatment.

### Pentamidine inhibits S100B-induced lipid peroxidation, p38 MAPK phosphorylation, and NF-κB activation induced by DSS

**Figure 4:**
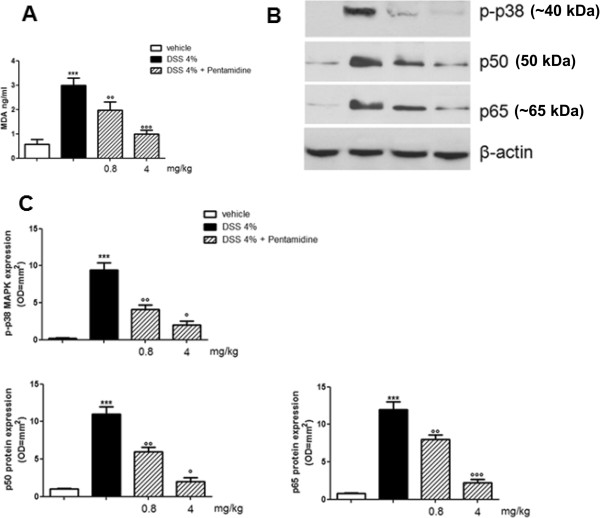
**(A) Effect of pentamidine on MDA production in colonic tissue of DSS-treated mice; (B) immunoblots showing the effect of pentamidine on p-p38 MAPK, p50 and p65 protein expression in colonic tissue of DSS-treated mice; (C) relative quantification of immunoreactive bands. **Results are expressed as mean ± SEM of *n* = 5 experiments performed in triplicate. ***P <0.001 vs. vehicle (saline); °*P *<0.05, °°*P *<0.01 and °°°*P *<0.001 vs. DSS. DSS, dextran sodium sulphate; MDA, malondialdehyde; p-p38 MAPK, phosphorylated-p38 MAPkinase.

Further characterizing the inhibitory effect of pentamidine, we found that in DSS-treated mice the increase in S100B expression was accompanied by a significant increase in lipid peroxidation and phosphorylation of p38 MAPK (Figure 4A and B). These effects were related to both p50 and p65 protein overexpression and indicated the activation of NF-κB (Figure 4B). In our experimental conditions, pentamidine significantly and dose-dependently inhibited MDA accumulation, p38 MAPK phosphorylation and, consequently, NF-κB activation.

### Pentamidine reduces DSS-induced macrophage infiltration and MPO activity

**Figure 5:**
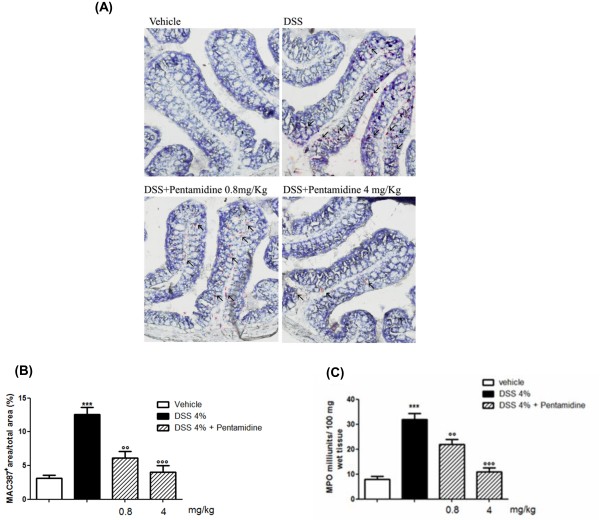
**(A) Effect of pentamidine on macrophage infiltration in colonic tissue of DSS-treated mice: pentamidine dose-dependently reduced macrophages infiltration.** Arrows indicate MAC387 immunopositive macrophages infiltrating colon criptae; **(B)** Quantification of MAC387 immunopositive macrophages in colon criptae. Data are expressed as mean ± SEM of *n* = 3 experiments; **(C) **effect of pentamidine on MPO level in colonic tissue of DSS-treated mice. Results are expressed as mean ± SEM of *n* = 6 experiments. ***P <0.001 vs. vehicle (saline); °°*P* <0.01 and °°°*P *<0.001 vs. DSS. Original magnification 100X. DSS, dextran sodium sulphate; MPO, mieloperoxidase.

Colonic mucosa was extensively infiltrated by macrophages in DSS-treated mice compared to control mice (Figure 5A and B). Such increase was accompanied as well by neutrophil infiltration, as showed by the increased MPO activity (Figure 5C). Pentamidine treatment caused a significant and dose-dependent reduction in macrophage infiltration together with significant and dose-dependent inhibition of MPO activity, indicating that pentamidine is able to control neutrophil infiltration in colonic tissues (Figure 5A and B).

### Pentamidine induces p53 expression in infiltrating macrophages

**Figure 6:**
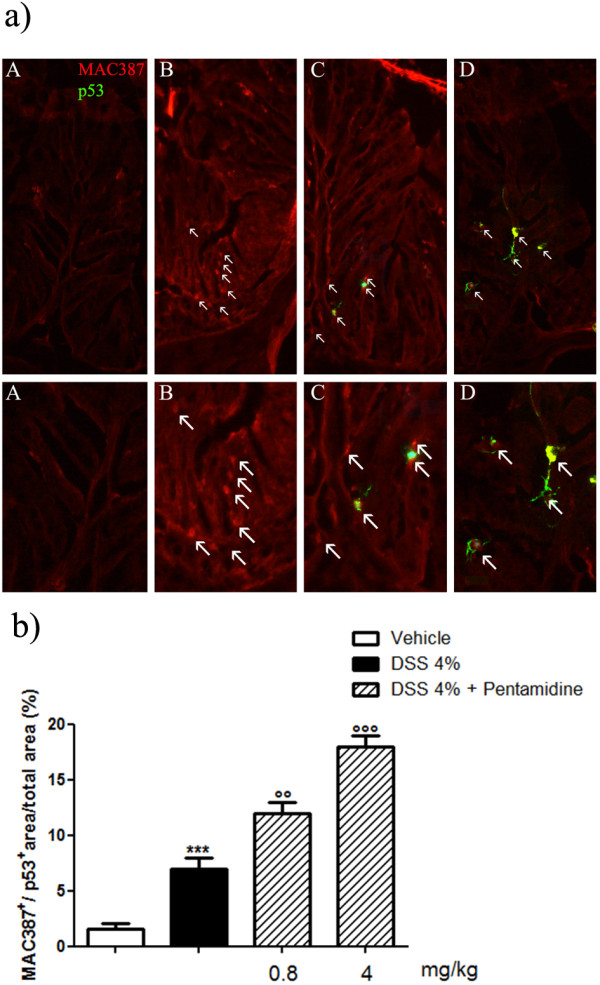
**(A) Immunofluorescence analysis of colonic tissues showing the effect of pentamidine on macrophage infiltration induced by DSS: pentamidine dose-dependently inhibited macrophage infiltration (MAC387, red) and increased p53 expression (green) in colon criptae. **Arrows indicate MAC387/p53 co-expression; **(B) **relative quantification (MAC387+/p53 + area/total area percentage) of macrophage infiltration and p53 expression in DSS-treated mice. Data are expressed as mean ± SEM of *n* = 3 experiments. Original magnification 200X. DSS, dextran sodium sulphate.

Macrophage infiltration in the colonic tissues from DSS-treated mice was confirmed by immunofluorence (Figure 6A). Pentamidine treatment significantly and dose-dependently reduced macrophage infiltration (Figure 6A). Very interestingly, such decrease was accompanied by the reverse trend in p53 expression. In fact, p53 resulted negative in the colon from control and DSS-treated mice, while a strong immunopositivity was found in colonic tissues from pentamidine-treated mice (Figure 6). Moreover, p53 expression was found in macrophages, as revealed from the co-expression with MAC387, a specific macrophage marker [21] (Figure 6).

### S100B-dependent effect of pentamidine

**Figure 7:**
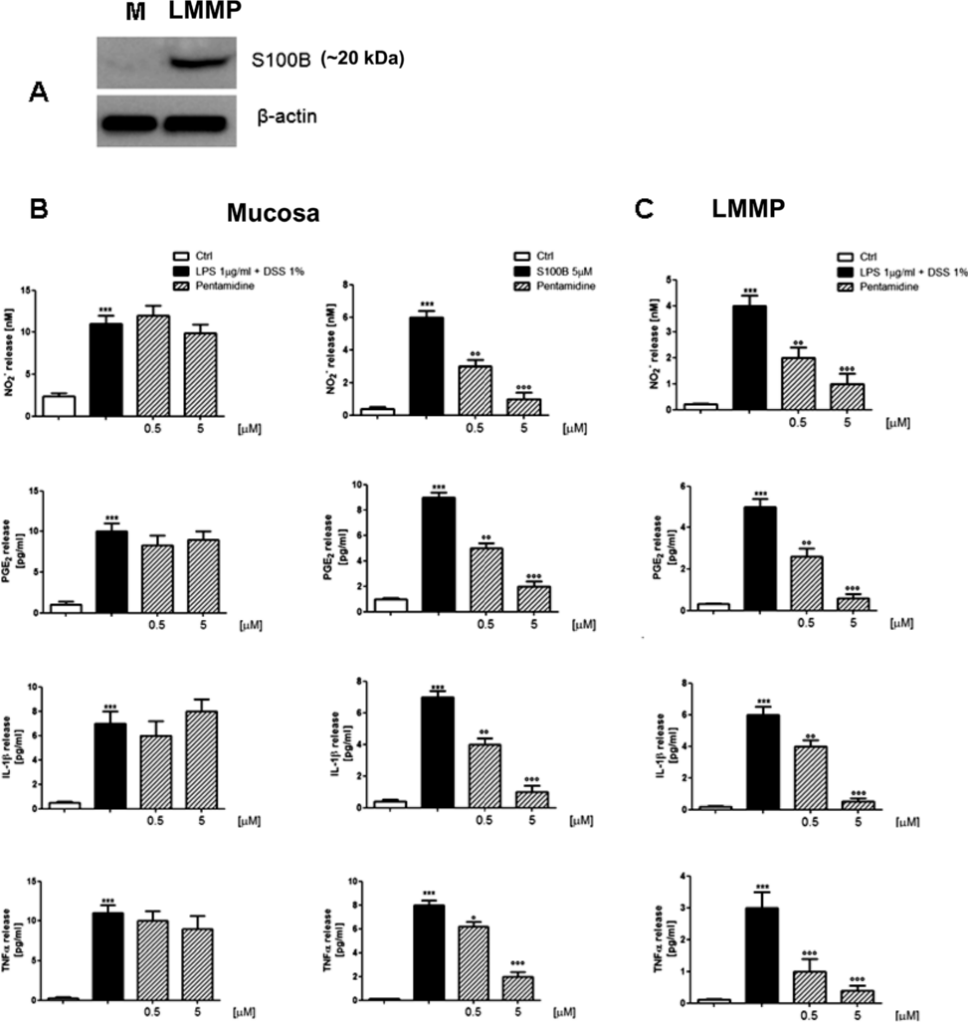
**(A) Western blot analysis showing S100B protein expression in mucosa (M) and in LMMP preparations. **Effect of pentamidine on inflammatory mediators (NO_2_^-^, PGE_2_, IL-1β, TNF-α) in mucosa **(B) **and in LMMP **(C) **in the presence of different proinflammatory stimuli (LPS + DSS or exogenous S100B depending upon the experiments). Results are expressed as mean ± SEM of *n* = 5 experiments. ***P <0.001 vs. vehicle (saline); °*P *<0.05, °°*P* <0.01 and °°°*P *<0.001 vs. proinflammatory stimulus DSS, dextran sodium sulphate; IL-1β, interleukin-1 beta; LMMP, longitudinal-muscle myenteric plexus; LPS, lipopolysaccharide; NO, nitric oxide; TNF-α, tumor necrosis factor alpha.

An internal check for the expression of S100B protein was performed in both mucosal and LMMP preparations confirming that the protein was expressed exclusively in the LMMP layer (Figure 7A). Preparations were separately challenged with DSS (1%) plus LPS (1 μg/ml) or S100B (5 μM) in the presence or absence of pentamidine (0.5 to 5 μM). Stimulation with LPS + DSS caused a marked proinflammatory response in the mucosa as well as in LMMP preparations, as demonstrated by the increase in nitrites level, PGE_2_ and TNF-α level compared to respective nonstimulated preparations (Figure 7B and C). Very interestingly, pentamidine had no significant beneficial effect on the mucosa while it significantly and concentration-dependently reduced the release of nitrites, PGE_2_ and TNF-α in LMMP preparations (Figure 7C). Exogenous application of S100B to the mucosa caused a significant increase in nitrites, PGE_2_, and TNF-α level (Figure 7B). As confirmation for the S100B-dependent effect, pretreatment with pentamidine significantly and concentration-dependently reduced S100B-induced inflammation in the mucosa (Figure 7C).

## Discussion

The results of the present study indicate that pentamidine is capable to profoundly and beneficially impact on an animal model of acute colitis. The model we used has been demonstrated to resemble UC features [18]. Prevailing therapies for UC include chronic administration of glucocorticosteroids and mesalamine [25,26]. Steroids are effective in the short-term treatment of acute flares of UC but they are not suitable as a maintenance therapy due to a variety of systemic adverse reactions [27]. Sulfasalazine and its derivative 5-aminosalicylic acid are effective only in mild-to-moderate acute phase of the disease and in preventing relapse. Biological drugs such as monoclonal anti-TNF-α antibody (infliximab and adalimumab) have been recently introduced in the therapy of relapsing inflammatory bowel disease with encouraging results in the maintenance of remission [28]. However, the long-term safety of these drugs, the possibility to induce severe side effects [29-31] together with the high costs of the therapy for the patients warrant novel and alternative pharmacological approaches.

We demonstrated that pentamidine efficiently and dose-dependently improved colitis. It caused attenuation of the DAI together with preservation of colonic length and reduction of splenomegaly induced by DSS-colitis. Besides inducing such macroscopic beneficial effects, treatment with pentamidine also resulted in microscopic amelioration of intestinal inflammation as demonstrated by the reduction of MPO activity, a marker of tissue neutrophils activation, and of macrophage mucosal infiltration. Very interestingly, we found that the treatment with pentamidine induced p53 expression on infiltrating macrophages. We know that S100B inhibits p53 activity and that pentamidine inhibits S100B overexpression. Thus, we may speculate that pentamidine, through the inhibition of S100B, induces p53 expression indirectly driving macrophages apoptosis. This trend could also explain the anti-inflammatory effect exerted by pentamidine.

As expected, DSS treatment caused a marked increase in COX-2 and iNOS protein expression in the colon accompanied by a significant release of PGE_2_ and NO in the plasma. Such increase was significantly and dose-dependently reduced by pentamidine treatment. We previously demonstrated that during intestinal inflammation, S100B rises up to micromolar concentrations, further amplifying inflammatory responses [6,8,12]. In these conditions, S100B induces lipid peroxidation and activates p38 MAPK phosphorylation leading, in turn, to NF-κB activation and proinflammatory cytokines release. Here again, we report that pentamidine treatment resulted in a significant inhibition of lipid peroxidation, p38MAPK activation and relative stabilization of NF-κB in the cytoplasm by targeting S100B activity. These results further confirm that pentamidine, by interfering at the p53 binding site on S100B protein, drastically reduces inflammatory events induced by S100B, leading to the overall improvement of intestinal inflammation.

Given the well-described inhibitory effect exerted by pentamidine on S100B activity [17], we evaluated whether it could negatively control enteric gliosis, thus exerting a negative modulation of intestinal inflammation. The administration of DSS demonstrated an enormous increase of both S100B and GFAP protein expression. Pentamidine was not capable of negatively modulating S100B protein expression in tissues and its release in plasma, while it was able to induce a significant decrease in GFAP protein expression. This data could be explained by the fact that since pentamidine inhibits the activity but not the expression/release of S100B [17], this drug may prevent the downstream effects due to the over-release of S100B during intestinal inflammation. In an autocrine manner, S100B induces proliferation of glial cells and, thus, increase in GFAP expression. We can thus speculate that pentamidine, by the inhibition of S100B activity, may control glial proliferation as highlighted by the decrease in GFAP expression. Alternatively, the ability of pentamidine to significantly reduce the expression of GFAP may be secondary to the reduced levels of inflammatory cytokines that have been previously demonstrated to drive glial proliferation [32].

Since S100B release has been reported to increase macrophage activity causing proinflammatory responses [33], we wondered whether the anti-inflammatory effect exerted by pentamidine on acute intestinal inflammation could be the result of the direct targeting of S100B in the glial network or the consequence of the indirect effect on infiltrating immune cells in the mucosa. We used organotypic cultures of isolated mucosa or LMMP preparations [24] and we challenged them with DSS plus LPS in the presence or absence of pentamidine. Our results demonstrated that pentamidine was able to significantly and concentration-dependently inhibit LPS + DSS-induced NO_2_^-^, PGE_2_, IL-1β and TNF-α release in LMMP but not in mucosal preparations. This result suggests that pentamidine needs S100B to exert its anti-inflammatory properties. To confirm such hypothesis, we challenged mucosa preparations with exogenous S100B in the presence or absence of pentamidine. Interestingly, we found that pentamidine significantly reversed S100B-induced release of inflammatory mediators, thus demonstrating that anti-inflammatory properties of pentamidine are dependent on S100B.

## Conclusions

Pentamidine may be putatively proposed as a new drug able to control the acute phase of intestinal inflammation, likely by acting on glial activation and thus inhibiting the deleterious cascade induced by S100B protein. Because of its well-known pharmacological and toxicological profile, at present, pentamidine might be regarded as a potential, innovative, manageable, and low-cost tool against colitis.

## Abbreviations

COX2: cyclooxigenase-2; DAI: disease activity index; DSS: dextran sodium sulphate; EGC: enteric glial cells; GFAP: glial fibrillary acidic protein; IL-1β: interleukin-1 beta; iNOS: inducible nitric oxide synthase; LMMP: longitudinal muscle-myenteric plexus; LPS: lipopolysaccharide; MDA: malonyldialdehyde; MPO: mieloperoxidase; NF-κB: nuclear factor-kappaB; NO: nitric oxide; PGE2: prostaglandin E2; p-p38 MAPK: phosphorylated-p38 MAPkinase; RAGE: receptor for advanced glycation end products; TNF-α: tumor necrosis factor alpha; UC: ulcerative colitis.

## Competing interests

All authors declare that they have no competing interest.

## Authors’ contribution

GE and EC conceived the study; GE designed experiments; EC performed experiments; GS and CC analyzed the data and contributed to the critical revision of the research plan; CS collected the data. GE, LS and RC equally contributed to the design of the study and writing and final approval of the manuscript. All authors read and approved the final manuscript.
